# Supplementary material for: A molnupiravir-associated mutational signature in global SARS-CoV-2 genomes
Source: Nature. 2023 Sep 25;623(7987):594–600. doi: 10.1038/s41586-023-06649-6 (PMC10651478; doi:10.1038/s41586-023-06649-6)
Supplement: Supplementary file 2 — Reporting Summary [file 41586_2023_6649_MOESM2_ESM.pdf]

nature portfolio

Corresponding author(s): Theo Sanderson

Last updated by author(s): Feb 9, 2023

Reporting Summary

Nature Portfolio wishes to improve the reproducibility of the work that we publish. This form provides structure for consistency and transparency in reporting. For further information on Nature Portfolio policies, see our Editorial Policies and the Editorial Policy Checklist.

Statistics

For all statistical analyses, confirm that the following items are present in the figure legend, table legend, main text, or Methods section.

n/a	Confirmed	
☐	☒	The exact sample size (*n*) for each experimental group/condition, given as a discrete number and unit of measurement
☒	☐	A statement on whether measurements were taken from distinct samples or whether the same sample was measured repeatedly
☐	☒	The statistical test(s) used AND whether they are one- or two-sided<br>*Only common tests should be described solely by name; describe more complex techniques in the Methods section.*
☐	☒	A description of all covariates tested
☐	☒	A description of any assumptions or corrections, such as tests of normality and adjustment for multiple comparisons
☐	☒	A full description of the statistical parameters including central tendency (e.g. means) or other basic estimates (e.g. regression coefficient) AND variation (e.g. standard deviation) or associated estimates of uncertainty (e.g. confidence intervals)
☐	☒	For null hypothesis testing, the test statistic (e.g. *F*, *t*, *r*) with confidence intervals, effect sizes, degrees of freedom and *P* value noted<br>*Give P values as exact values whenever suitable.*
☒	☐	For Bayesian analysis, information on the choice of priors and Markov chain Monte Carlo settings
☒	☐	For hierarchical and complex designs, identification of the appropriate level for tests and full reporting of outcomes
☒	☐	Estimates of effect sizes (e.g. Cohen's *d*, Pearson's *r*), indicating how they were calculated

Our web collection on statistics for biologists contains articles on many of the points above.

Software and code

Policy information about availability of computer code

Data collection	Code was not used to collect data in this study.
Data analysis	We have made a version of our analysis that uses a fully open dataset (therefore covering with fewer sequences) and open source code available at https://github.com/theosanderson/molnupiravir . This is available at Zenodo at https://zenodo.org/record/8309773 .<br><br>Chronumental v0.0.60 was used<br>Nextclade v2.12.0 was used<br>BTE v0.9.0 was used<br>MutTui v2.0.2 was used<br><br>Full R package versions available at https://github.com/theosanderson/molnupiravir/blob/main/archive/package_versions.txt

For manuscripts utilizing custom algorithms or software that are central to the research but not yet described in published literature, software must be made available to editors and reviewers. We strongly encourage code deposition in a community repository (e.g. GitHub). See the Nature Portfolio guidelines for submitting code & software for further information.

Data

Policy information about availability of data

All manuscripts must include a data availability statement. This statement should provide the following information, where applicable:

- Accession codes, unique identifiers, or web links for publicly available datasets
- A description of any restrictions on data availability
- For clinical datasets or third party data, please ensure that the statement adheres to our policy

No new primary data was generated for this study. We used data from consensus sequences available through GISAID and the INSDC24,25, from the AGILE clinical trial 20, where genomic data were obtained from BioProject PRJNA854613 at the SRA, and from Alteri et al 18 from BioProject ERP142142. The AGILE investigators were not involved in the analysis and preparation of this manuscript. Linkage analysis was performed within UKHSA. Section 251 of the National Health Service Act 2006 permits UKHSA use of patient-level data for specific projects. The findings of this study are based on metadata associated with 15,572,413 sequences available on GISAID up to June 2023, and accessible at 10.55876/gis8.230110wz, and 10.55876/gis8.230110db, 10.55876/gis8.230622mw (see also, Supplemental Tables). The findings of this study are also based on 7,104,124 sequences from INSDC.

We analysed these data in the form of a mutation-annotated tree, which is a version of McBroome et al. Data present in both databases are deduplicated during the construction of the mutation-annotated tree on the basis of sequence, name, and metadata. We standardised to GISAID sequence names and accessions for sequences present in both databases.

A version of our analysis using only the INSDC subset of the tree, with INSDC naming conventions, is available at https://github.com/theosanderson/molnupiravir/tree/main/open_data_version. Processed open data is available at https://zenodo.org/record/8252388 .

Human research participants

Policy information about studies involving human research participants and Sex and Gender in Research.

Reporting on sex and gender	N/A
Population characteristics	N/A
Recruitment	N/A
Ethics oversight	N/A

Note that full information on the approval of the study protocol must also be provided in the manuscript.

Field-specific reporting

Please select the one below that is the best fit for your research. If you are not sure, read the appropriate sections before making your selection.

☒ Life sciences ☐ Behavioural & social sciences ☐ Ecological, evolutionary & environmental sciences

For a reference copy of the document with all sections, see nature.com/documents/nr-reporting-summary-flat.pdf

Life sciences study design

All studies must disclose on these points even when the disclosure is negative.

Sample size	We used all available data
Data exclusions	No data were excluded
Replication	This study used all available data. We replicate results in the sense that similar effects are seen in multiple different countries that use molnupiravir. We did not conduct experiments but analysed data that was available in databases.
Randomization	This study did not have experimental groups. We analysed all available data. There was no opportunity for randomization.
Blinding	No blinding was performed. Blinding was not necessary given the large number of sequences being analysed and the limited degrees of freedom available.

Reporting for specific materials, systems and methods

We require information from authors about some types of materials, experimental systems and methods used in many studies. Here, indicate whether each material, system or method listed is relevant to your study. If you are not sure if a list item applies to your research, read the appropriate section before selecting a response.

Materials & experimental systems

n/a	Involved in the study
☒	☐ Antibodies
☒	☐ Eukaryotic cell lines
☒	☐ Palaeontology and archaeology
☒	☐ Animals and other organisms
☒	☐ Clinical data
☒	☐ Dual use research of concern

Methods

n/a	Involved in the study
☒	☐ ChIP-seq
☒	☐ Flow cytometry
☒	☐ MRI-based neuroimaging
